# Genotype imputation in case-only studies of gene-environment interaction: validity and power

**DOI:** 10.1007/s00439-021-02294-z

**Published:** 2021-05-26

**Authors:** Milda Aleknonytė-Resch, Silke Szymczak, Sandra Freitag-Wolf, Astrid Dempfle, Michael Krawczak

**Affiliations:** 1 Institute of Medical Informatics and Statistics, Kiel University, Kiel, Germany; 2 Institute of Medical Biometry and Statistics, University of Lübeck, Lübeck, Germany

## Abstract

Case-only (CO) studies are a powerful means to uncover gene-environment (G × E) interactions for complex human diseases. Moreover, such studies may in principle also draw upon genotype imputation to increase statistical power even further. However, genotype imputation usually employs healthy controls such as the Haplotype Reference Consortium (HRC) data as an imputation base, which may systematically perturb CO studies in genomic regions with main effects upon disease risk. Using genotype data from 719 German Crohn Disease (CD) patients, we investigated the level of imputation accuracy achievable for single nucleotide polymorphisms (SNPs) with or without a genetic main effect, and with varying minor allele frequency (MAF). Genotypes were imputed from neighbouring SNPs at different levels of linkage disequilibrium (LD) to the target SNP using the HRC data as an imputation base. Comparison of the true and imputed genotypes revealed lower imputation accuracy for SNPs with strong main effects. We also simulated different levels of G × E interaction to evaluate the potential loss of statistical validity and power incurred by the use of imputed genotypes. Simulations under the null hypothesis revealed that genotype imputation does not inflate the type I error rate of CO studies of G × E. However, the statistical power was found to be reduced by imputation, particularly for SNPs with low MAF, and a gradual loss of statistical power resulted when the level of LD to the SNPs driving the imputation decreased. Our study thus highlights that genotype imputation should be employed with great care in CO studies of G × E interaction.

**Supplementary Information:**

The online version contains supplementary material available at 10.1007/s00439-021-02294-z.

## Introduction

The genetic aetiology of most common complex diseases such as, for example, cancer, diabetes and asthma is still poorly understood. General progress in this direction has been hampered by the fact that the diseases in question result from a large number of genetic and environmental factors, each with only a small effect upon disease risk. The consequent causative complexity is exacerbated further by a number of related phenomena (Manolio et al. 2009) including gene–gene (G × G) and gene-environment (G × E) interaction, among others.

The precise meaning of ‘interaction’ depends upon the context in which this expression is being used as either a biological or a statistical term (Cordell 2002; Dempfle et al. 2008). Biological interaction usually refers to the combined effect of two causal factors that interact physically or chemically, or that affect the same disease-relevant biological pathway (Yang and Khoury 1997). Statistical interaction, by contrast, is defined as the “departure from additivity of effects on a specific outcome scale” (Rothman et al. 2008). It is tantamount to so-called ‘effect modification’, meaning that the risk difference associated with one factor on a specific scale depends upon the presence or absence of the other risk factor. In the following, we will focus upon the statistical interaction of two risk factors on the logit scale, i.e. we shall deal with departures from the multiplicity of the corresponding odds ratios (OR). Ideally, statistical interaction points towards plausible biological interaction, but the two need not necessarily coincide (Cowman and Koyutürk 2017).

Genetic epidemiological studies of common complex diseases employ different designs and methods, and the case-control (CC) design has become the ‘work horse’ in this context, particularly in the form of genome-wide association studies (GWAS) of single nucleotide polymorphisms (SNPs). For studies of G × E interaction, however, the case-only (CO) design has also received some attention (Piegorsch et al. 1994) because it provides several advantages over the CC design (Gauderman 2002a; Kraft et al. 2007). First and foremost, only cases (i.e. patients affected by the disease of interest) are required, which obviates the often difficult identification and recruitment of suitable controls (Schulz and Grimes 2002). Second, the CO study design entails a substantial gain in statistical power to detect G × E over a CC design using the same number of cases (Gauderman 2002a). On the other hand, however, the reliability of CO studies of G × E hinges upon the validity of two critical assumptions, namely (i) that the disease of interest is sufficiently rare (i.e. has prevalence ≤ 5%, say) and (ii) that the two risk factors under study (genetic and environmental) are uncorrelated in the general population (Piegorsch et al. 1994). Although the last presumption may often seem justified, it still needs to be reviewed carefully for each study. For example, some variants in genes associated with alcohol metabolism are known to be linked to alcohol consumption (Goldman et al. 2005), and even such minor gene-environment associations can lead to false-positive results in CO studies of G × E (Albert 2001).

For some time, researchers studying the genetic basis of common complex disease have been trying to improve the inferential capacity of GWAS through genotype imputation (Marchini and Howie 2010). With this technique, genotypes of untyped SNPs are predicted from the genotypes of typed SNPs in a panel of reference haplotypes by way of exploiting population-level linkage disequilibrium (LD). Genotype imputation has since become a standard for GWAS because it facilitates the harmonization of SNP panels, improves statistical power by increasing sample size, and allows greater genomic coverage in terms of the number and density of the SNPs considered (Naj 2019). Genotype imputation can be performed either offline or using a web-based service such as the Michigan Imputation Server (Das et al. 2016) or the Sanger Imputation Server (The Haplotype Reference Consortium 2016). Notably, all software available for imputation provides means to assess the quality of the predicted genotypes, usually through the provision of an imputation quality score. The Michigan Imputation Server used in the present study, for example, generates an ﻿*﻿R*^﻿2^ quality score that relates the empirical variance of the imputed genotypes to its expectation at Hardy–Weinberg equilibrium (Das et al. 2016).

Irrespective of its potential to improve inferential capacity, genotype imputation is still an error-prone technique that can cause bias in subsequent analyses. Thus, the presence of hidden population stratification, the use of an inappropriate imputation base, and a lack of sufficient SNP coverage may all negatively affect imputation quality (Zhang et al. 2011; Das et al. 2018; Schurz et al. 2019). Moreover, the imputation quality achievable in a certain GWAS may still vary substantially along the human genome (Naj 2019). On the other hand, misclassification of genotypes is known to cause spurious gene-environment associations in CC studies (Wong et al. 2004), and Cheng and Lin (2009) demonstrated how genotype misclassification can reduce the power of both the CC and the CO design.

We previously examined the impact of LD upon the validity of CO studies of G × E (Yadav et al. 2015b), and subsequently developed means to allow for hidden population stratification in such studies (Yadav et al. 2015a). Extending this earlier work, we here present an investigation of how genotype imputation accuracy influences the validity and power of CO studies of G × E, an aspect that to our knowledge has not been studied in detail so far. In our simulation-based study, we paid specific attention to the fact that in regions with genetic main effects upon disease risk, haplotype frequencies, and hence LD, are bound to differ systematically between cases and controls. We also considered different environmental exposure frequencies when simulating G × E interactions to cover a broad range of realistic GWAS scenarios.

## Methods

### Data

The main goal of our study was to determine under which conditions imputed genotypes still allow reliable inference of the presence and magnitude of G × E interaction, using a CO design. Even though our investigations were mostly simulation-based, we nevertheless chose to employ real SNP genotype data to ensure realistic LD patterns in our study samples (Kulle et al. 2005; Ramnarine et al. 2015). Systematically varying the parameters of interest, namely the G × E odds ratio (OR) and the environmental exposure frequency, individual exposure states were randomly assigned to individuals according to their given SNP genotypes.

The data, which comprised 719 Crohn disease (CD) patients and 2491 healthy controls from Northern Germany, were kindly provided to us by the PopGen biobank (Krawczak et al. 2006). All individuals had been genotyped before for 156 k SNPs, covering all human autosomes. The genotypes coincided with the ‘Germany, Kiel’ set used by Yadav et al. (2017) in their global study of gene-smoking interactions, which means that the data underlying the present study had been subject to the quality control measures of the earlier study.

### Analysis strategy

Our analysis comprised two tiers: In tier 1, we first masked the genotypes of selected SNPs (henceforth referred to as ‘target SNPs’) in the cases and imputed them from a suitable imputation base (see below). Next, we compared the minor allele frequencies (MAFs) of the true and imputed SNP genotypes (i.e. allele dosages) both to one another and to the MAFs of the European population of the Haplotype Reference Consortium (The Haplotype Reference Consortium 2016; HRC), which served as an imputation base in our study. Finally, the imputed allele dosages were compared to the true genotypes using Cohen’s kappa to quantify the level of genotype concordance for each SNP. In tier 2, we simulated binary environmental exposure states (1: exposed, 0: non-exposed) for the cases depending upon the presumed G × E odds ratio and the original genotype of the target SNP under study. Consideration of different types of target SNP allowed us to study the effects of genotype imputation upon the validity of subsequent G × E interaction analyses under different scenarios regarding MAF, main effect OR and surrounding LD structure.

All statistical analyses were carried out with R (v. 3.5.0) or PLINK2 (Chang et al. 2015). For statistical modelling, SNP genotypes (G) were encoded assuming a dominant G × E effect of the minor allele, i.e. G = 1 for homozygous or heterozygous carriers of the minor allele, G = 0 for homozygous carriers of the major allele. A simple dominant model was used because it can cover a wide range of plausible genotype-phenotype relationships (Guan et al. 2012).

### SNP selection

The choice of SNPs for tier 1 (i.e. the genotype imputation) was based upon the respective MAF and the presence or absence of a main effect on CD risk. To this end, the disease ORs of SNPs were determined by way of a CC logistic regression association analysis of all SNPs that passed quality control, adjusted for the first 10 principal components of these SNPs to allow for possible population stratification (Price et al. 2006). SNPs with a disease association *p* value < 10^–5^ were considered further and pruned according to the following pair-wise criteria: (i) main effect OR difference ≤ 0.02, (ii) MAF difference in cases ≤ 0.02, and (iii) physical distance ≤ 15 kb. Pruning left 141 ‘independent’ SNPs that were grouped into four MAF-defined categories: low (MAF < 0.05), medium low (0.05 ≤ MAF < 0.15), medium high (0.15 ≤ MAF < 0.25) and high (0.25 ≥ MAF). All main effect SNPs in the low (*n* = 16) and medium low (n = 7) category were included in the subsequent imputation, together with 18 SNPs per category randomly chosen from ‘medium high’ and ‘high’. These 59 main effect SNPs were complemented by 59 randomly selected SNPs lacking a main effect, chosen according to the following matching criteria: (i) localization on the same chromosome as the respective main effect SNP, and (ii) a MAF difference ≤ 0.01 in cases. A detailed list of the 118 target SNPs is provided in Supplementary Table 1.

Only target SNPs with a proven main effect were forwarded to tier 2 of our study (i.e. G × E simulation and analysis). Here, however, we excluded main effect SNPs with an imputed MAF < 0.005 or a missing rate > 0.2, bringing the final number of target SNPs in tier 2 to 56. These criteria were found to be sufficient to ensure that no segmentation errors occurred with the PLINK software in tier 2.

### Imputation (Tier 1)

Genotype imputation was carried out for the 118 target SNPs in 10 successive rounds, each time thinning further the set of SNPs driving the imputation. In the first round, only the genotypes of the 118 target SNPs themselves were masked; in the nth subsequent round (*n* = 2 to 10), the surrounding SNPs were LD-pruned maintaining only SNPs with r^2^ < (11-n)/10 to the target SNP. A script published by the Wellcome Centre for Human Genetics, Oxford, UK (https://www.well.ox.ac.uk/~wrayner/tools/) was used to prepare the datasets for genotype imputation with the Michigan Imputation Server (https://imputationserver.sph.umich.edu/), which uses minimac4, selecting Quality control and Eagle v. 2.4 phasing settings. The HRC European data comprising 39.6 Million SNP genotypes from each of 32,470 individual samples (The Haplotype Reference Consortium 2016) served as the imputation base.

### Simulation of G × E interaction (Tier 2)

For the simulation and analysis of G × E interaction, imputed genotypes were converted to hard-calls using a hard-call threshold of 0.8. Environmental exposure states were simulated assuming two different population-level exposure frequencies, namely 10% and 30%. Under a dominant model, G × E interaction manifests in cases through a different exposure frequency for carriers and non-carriers of the minor SNP allele. Hence, we simulated G × E interaction by assigning environmental exposure states to individuals depending upon their respective (true) SNP genotype. The necessary genotype-specific exposure probabilities were calculated in two steps: First, QUANTO software (Gauderman 2002b) was used for each of the 56 main effect target SNPs to calculate the G × E interaction OR that would be detectable with 80% statistical power, taking into account the main effect OR and MAF of the SNP, the population-level exposure frequency and the case sample size (*n* = 719). In the power calculations, a nominal significance level of 0.05 was assumed. QUANTO also requires a main effect OR for the environmental exposure, which was consistently set to 1.5. A summary of the resulting SNP genotype-specific exposure probabilities is provided in Supplementary Table 2. The null hypothesis of no G × E interaction, where all genotype-specific exposure probabilities equal the population-level value, was also simulated for each SNP to complement the G × E interaction analyses.

For each of the 56 main effect target SNPs, 10,000 replicates of the environmental exposure simulation were undertaken and the G × E interaction ORs determined for the 10 imputed genotype sets from tier 1. Since our study was concerned with a CO design, the G × E interaction OR was estimated from the simulated exposure and either the true or the imputed genotype data by logistic regression via

$${\text{logit}}\left\{ {P\left( {E = 1} \right)} \right\} = \beta _{0} + ~\beta _{1} G.$$

The statistical significance (i.e. the *p* value) of β_1_ ≠ 0 was determined for each replicate with a Wald test, and the overall statistical power was estimated by the proportion of replicates with *p* < 0.05. Following Piergosch et al. (1994), no classical confounders such as age or sex were included in the regression model because their main effects cannot sensibly be modelled in a CO design.

## Results

**Fig. 1:**
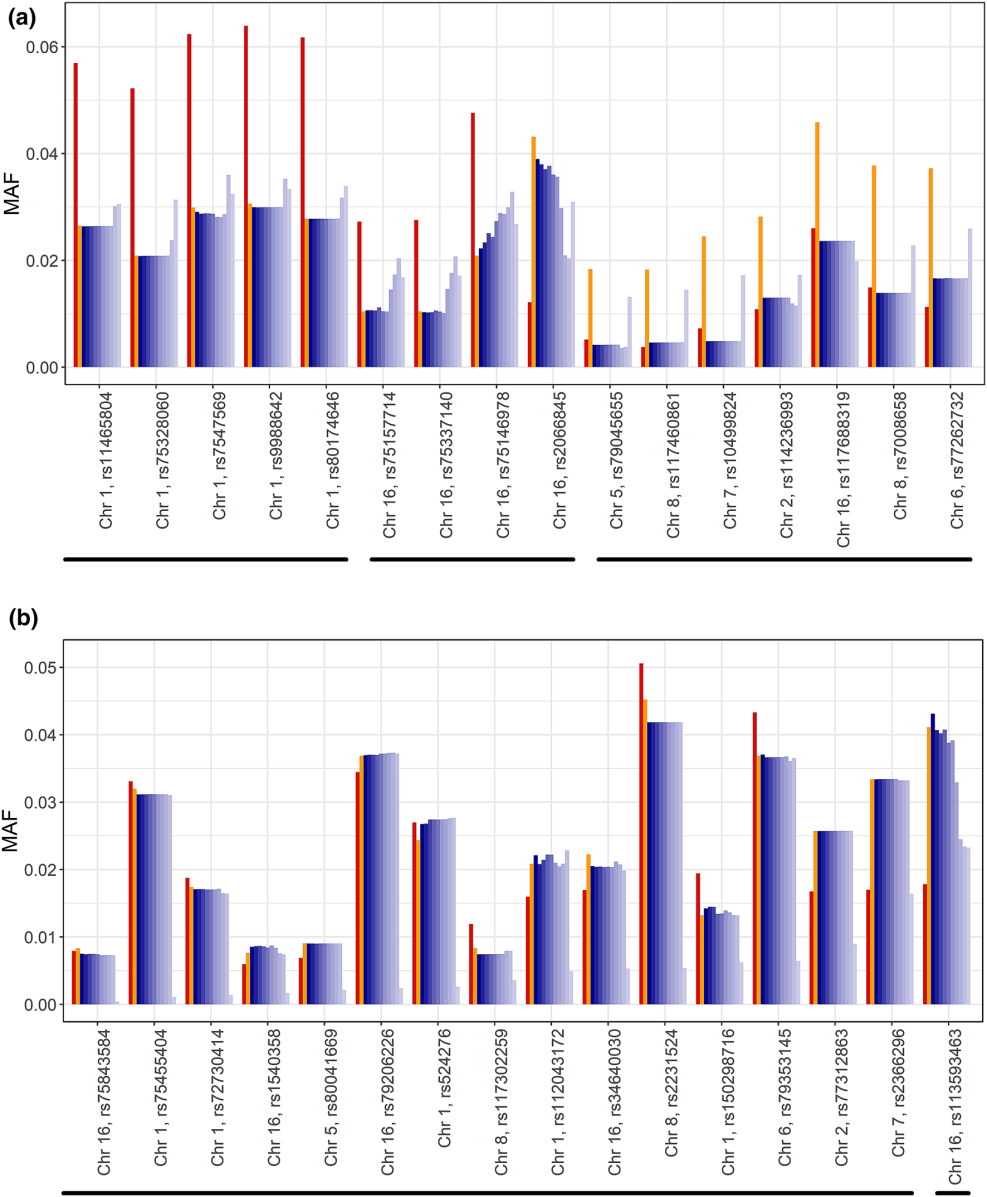
MAF of true and imputed genotypes of SNPs in the low MAF category (MAF < 0.05). Target SNPs are distinguished according to whether they (**a**) had a main effect on CD risk, i.e. showed a significant association with CD in the study data, or (**b**) lacked a main effect. The left-most bar (red) depicts the MAF in the HRC European population sample that served as the imputation base. The next bar (orange) marks the real MAF of the SNP in the CO sample. The adjacent bars (shades of blue) depict the MAF at advancing levels of LD pruning of the SNPs surrounding the target SNP, namely from *r*^2^ < 1.0 to *r*^2^ < 0.1 in steps of 0.1 (for details, see “Methods”. Grouping of SNPs by MAF characteristics, as referred to in the main text, is indicated by horizontal black bars

A first impression of the accuracy of SNP genotype imputation can be gained from the correspondence, or not, between the MAF of real and imputed genotypes. In this regard, our study of 118 target SNPs revealed that imputation seems to work well in a CO sample for SNPs lacking a main effect on disease risk, but less so when a main effect is present. Although this difference was most evident in the low MAF category of SNPs (MAF < 0.05; Fig. 1), it also became apparent in the other three categories (Supplementary Figs. 1, 2, 3).

For some main effect SNPs with low MAF, namely rs11465804 to rs80174646 (from left to right in Fig. 1a), the real and imputed MAF agreed well, irrespective of the regional level of LD of SNPs surrounding the target SNP. The MAF only changed when LD pruning was nearly complete. For SNPs rs75157713 to rs2066845 (Fig. 1a), the two MAFs agreed well initially but differed increasingly as LD pruning progressed. Concurrently, the imputed MAF was found to converge towards the MAF observed in the imputation base. For the remaining SNPs in the low MAF category, namely rs79045655 to rs77262732 (Fig. 1a), the real and imputed MAF were rather different right from the start, and the imputed MAF consistently resembled the MAF in the imputation base. Notably, the real MAF differed substantially from the MAF in the imputation base for all 16 main effect SNPs, thereby highlighting the fact that owing to their association with CD, the genotype distribution of these SNPs in cases would have been systematically different from that in an appropriate population reference in the first place.

For SNPs lacking a main effect on CD risk, a comparison between the true and imputed MAF revealed only minor differences in the low MAF category, with the sole exception of rs113593463 (Fig. 1b). Moreover, the imputed MAF was less responsive to LD pruning of the neighbourhood of the target SNP, and both MAFs resembled the MAF observed in the imputation base more closely than was the case for main effect SNPs.

**Fig. 2:**
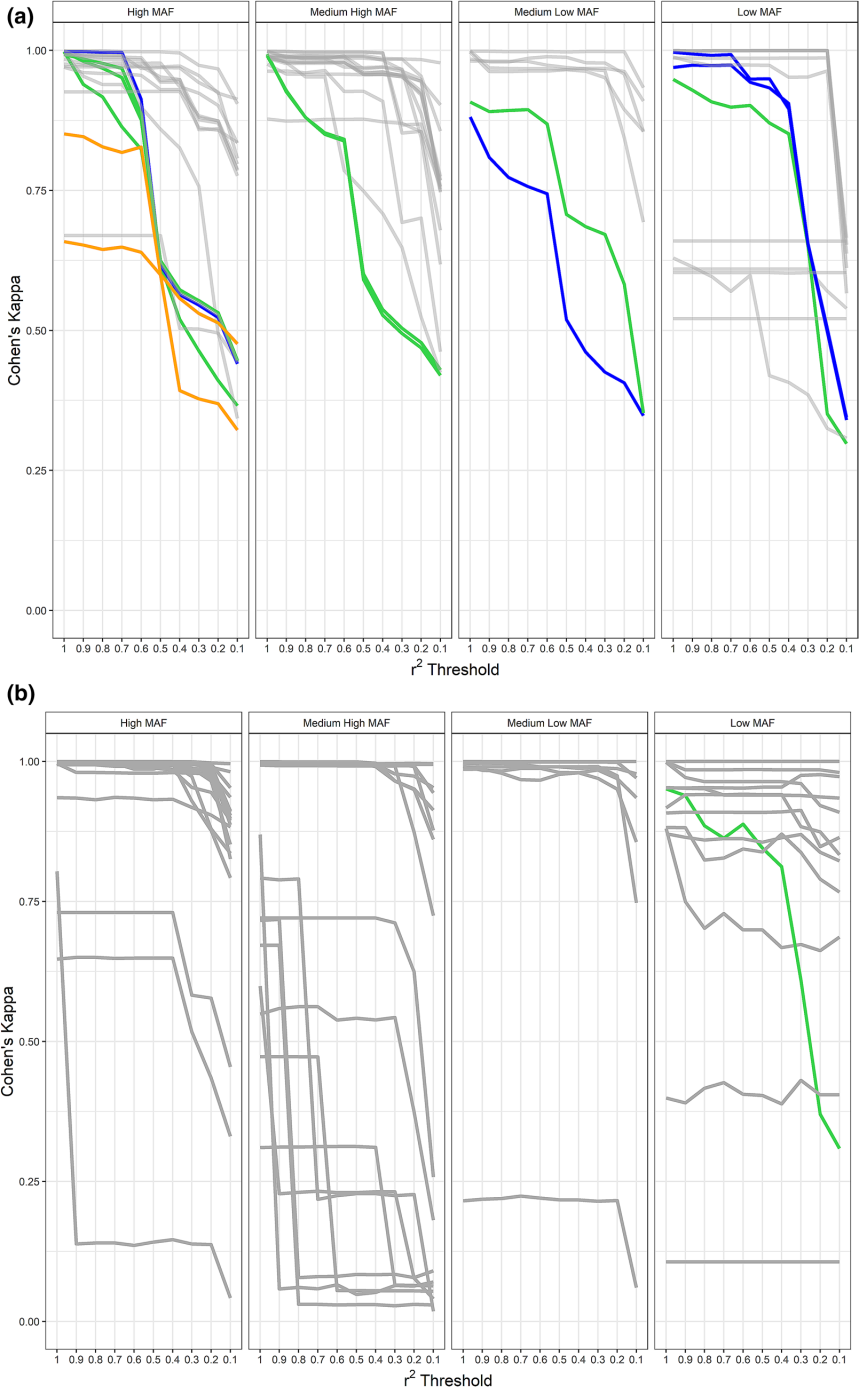
Cohen’s kappa of genotype agreement (real *vs* imputed) for target SNPs (**a**) with and (**b**) without a main effect on CD risk. Horizontal axis: LD pruning around each target SNP proceeded using an *r*^2^ threshold that varied between 1.0 (weak pruning) and 0.1 (strong pruning). SNPs in selected gene regions are color-coded (*NOD2*: green, *NKD1*: orange, *CYLD*: blue). For the definition of MAF categories of SNPs, see “Methods”

The trend towards poorer genotype imputation for main effect SNPs evidenced by the MAF differences also became apparent when the concordance of individual genotypes (real *vs* imputed) was quantified by Cohen’s kappa (Fig. 2). Except for a few outliers, particularly in the medium high MAF category, SNPs with a main effect on CD risk yielded lower kappa values (Fig. 2a) than SNPs lacking a main effect (Fig. 2b). Moreover, the main effect SNPs also showed a stronger decline in imputation accuracy when LD pruning progressed from weak (*r*^2^ < 1.0) to strong (*r*^2^ < 0.1). This effect was most prominent for SNPs in genomic regions known to be strongly associated with CD risk, namely around the *NOD2*, *NKD1* and *CYLD* genes (Cleynen et al. 2014). By contrast, Cohen’s kappa appeared to be less sensitive to LD pruning for SNPs without a main effect (Fig. 2b). An exception to this rule seemed to be rs113593463 from the low MAF category. However, this SNP is located in the *NOD2* gene region (highlighted by green colouring in Fig. 2b) and was characterized in our study data by a genotype–phenotype association *p* value of 3.3 × 10^–4^ (main effect OR: 1.82). Although not formally counting as a main effect SNP itself, genotype imputation was therefore likely hampered for rs113593463 by its proximity to other main effect SNPs in the same region.

**Fig. 3:**
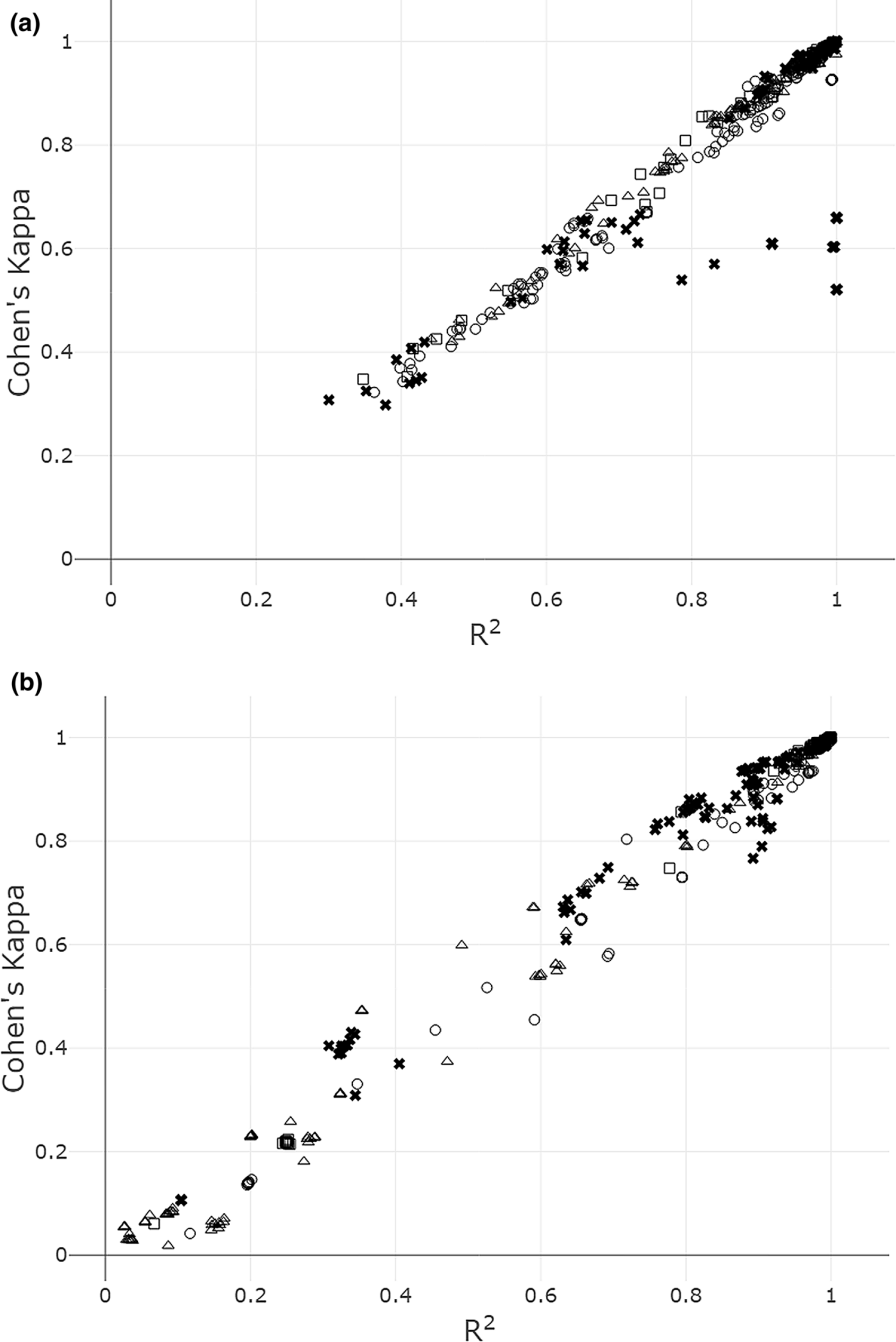
Estimated imputation accuracy score (*R*^2^ from minimac4) and Cohen’s kappa for target SNPs (**a**) with and (**b**) without a main effect on CD risk. MAF categories are labelled as follows: crosses (low), squares (medium low), triangles (medium high), circles (high)

Most imputation software, including the Michigan Imputation Server, provides some kind of SNP-specific ‘imputation accuracy score’ as a measure of imputation quality. Accuracy scores > 0.8 are usually considered adequate and conservative enough (Verma et al. 2014) for imputed genotypes to be used in downstream analyses. In view of this, scores in the named range should reflect good matching of true and imputed genotypes. Indeed, our analysis broadly confirmed this supposition by a strong correlation between imputation accuracy score and Cohen’s kappa in all MAF categories of SNPs (Fig. 3), with one exception: For several main effect SNPs in the ‘low’ category, the imputation accuracy score from minimac4 exceeded 0.8, yet the agreement between true and imputed genotypes, as quantified by Cohen’s kappa, was low (Fig. 3a).

**Fig. 4:**
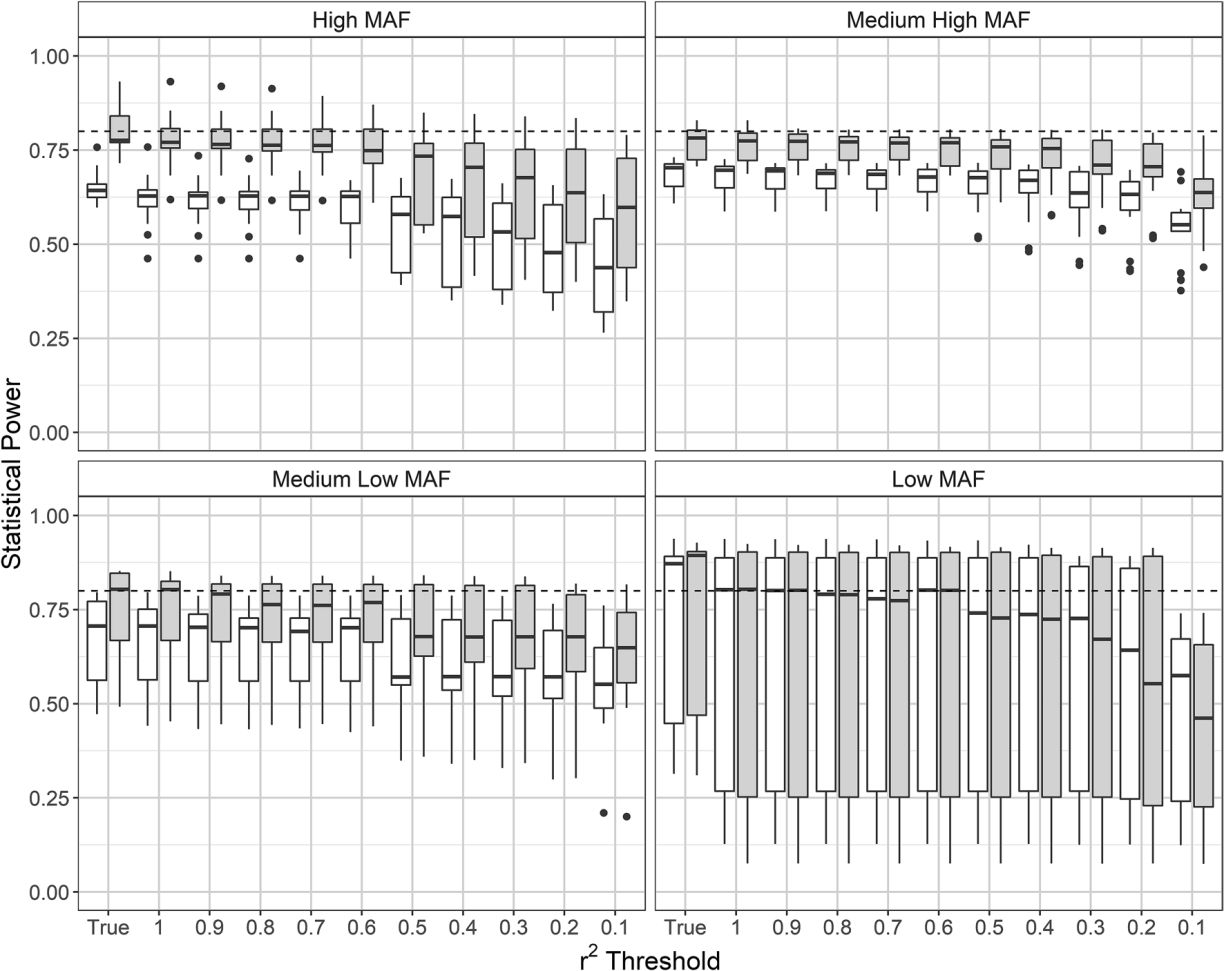
Simulation-based estimates of the statistical power to detect G × E interaction, using a CO design with imputed SNP genotypes (10,000 replicates per parameter setting). The interaction OR underlying the simulations was such that the true genotypes should have afforded 80% power according to the QUANTO software (depicted by a dashed line). Horizontal axis: *r*^2^ threshold used for LD pruning around the target SNP (for details, see “Methods” and legend to Fig. 2) except for column ‘True’, which refers to the non-imputed genotypes. Boxes are tinted according to the environmental exposure frequency underlying the simulations: grey (30%), white (10%). For the definition of MAF categories of SNPs, see “Methods”

Within the confines of resolution provided by the current sample size (*n* = 719), our simulations of an imputation-based G × E analysis using a CO study design revealed three main characteristics in terms of statistical power. First, a higher exposure frequency implies greater statistical power to detect a given level of G × E interaction (Fig. 4), an observation made in all but the low MAF category. In fact, when MAF ≥ 0.05, the median statistical power was consistently greater by at least 20% at an exposure frequency of 30% than at 10%, regardless of the LD pruning threshold employed. Second, while the median power appeared to be rather independent of the MAF, its variability was found to increase with decreasing MAF (Fig. 4). In the low MAF category, the interquartile range of the power thus spanned 0.25 to 0.80 except when LD pruning was strong (*r*^2^ < 0.1). Moreover, while the target SNP-specific G × E interaction ORs underlying our simulations should have consistently afforded 80% power to detect G × E with the true genotypes, this was obviously not the case when the exposure frequency was 10%. Finally, a gradual power loss occurred in all MAF categories when the neighbourhood of the target SNP was LD-pruned, with a prominent drop seen in the ‘high’ category between *r*^2^ thresholds 0.6 and 0.5.

**Fig. 5:**
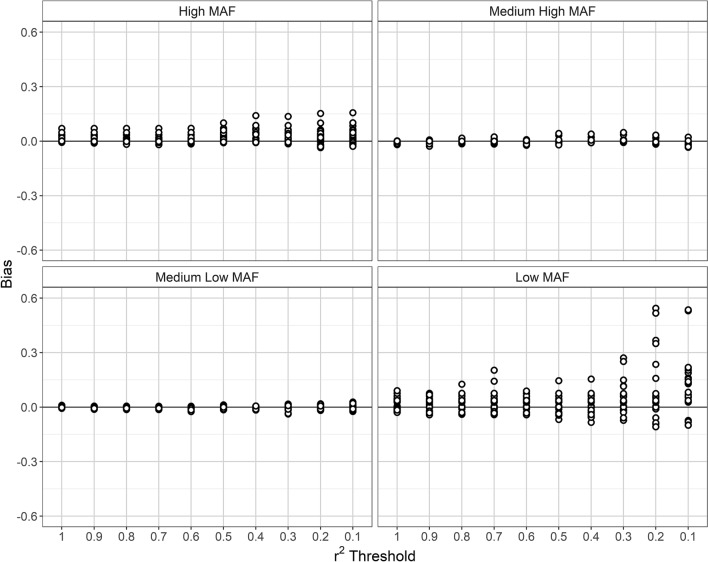
Bias of the estimated logistic regression coefficient β (i.e. logarithm of G × E interaction OR) at different levels of LD pruning. The bias was calculated as true genotype-based β minus imputation-based β

Our simulations also revealed that G × E interaction ORs estimates from imputed genotypes can be biased, particularly when the MAF of the target SNP is low (Fig. 5). This bias is the more prominent the more sparsely the genomic region of interest is saturated with SNPs driving the imputation.

Simulations were also carried out under the null hypothesis of no G × E interaction to assess the type I error rate of imputation-based CO studies. The results suggest that the type I error rate is not systematically inflated, regardless of the level of LD around the target SNP (Supplementary Figs. 4 and 5). On the contrary, as a flipside of its reduced power, the G × E analysis was even found to be overly conservative in the low MAF category in that the corresponding median type I error rate was consistently smaller than 0.05 (Supplementary Fig. 4). Finally, notable bias of the G × E interaction OR estimates was observed only in the low MAF category, and this bias was independent of the level of LD pruning (Supplementary Fig. 5).

## Discussion

Using real genotype data from over 700 German CD patients alongside simulated environmental exposure states, we studied how the power and validity of CO studies of G × E interaction are affected when the target SNP genotypes of the cases are imputed using population controls as an imputation base. We tried to cover realistic scenarios and therefore not only considered SNPs embedded into different real-world LD structures, and with different MAFs and main effects (ME) upon disease risk, but also systematically thinned out the respective imputation base so as to comprise fewer and fewer SNPs that could drive the imputation of the target SNP genotypes.

One of our main findings was that genotype imputation seems to be more prone to errors for target SNPs with an ME than for SNPs without an ME. This was exemplified by SNPs from the *NOD2*, *NKD1* and *CYLD* gene regions, all of which are known to harbour important risk factors for CD (Cleynen et al. 2014). Moreover, the imputation accuracy for ME SNPs was also found to depend strongly upon the amount of local LD that could be exploited for imputation. The correspondence between real and imputed genotypes, regardless of whether measured by MAF similarity, at the population level, or Cohen’s kappa, at the sample level, gradually deteriorated when the maximum LD between the target SNP and SNPs comprising the imputation base was artificially reduced. The difference noted in this regard between SNPs with and without an ME are plausible because the imputation base in our study comprised population controls, namely the HRC European data, which is unlikely to be representative of CD patients in genomic regions known to be associated with this disease. One target SNP formally lacking an ME (rs113593463) showed a similar decline in Cohen’s kappa as the ME SNPs, thereby providing a counter example at first glance. However, this SNP is located in the *NOD2* gene region and its disease association p value (3.3 × 10^–4^) just missed the significance threshold of 5 × 10^–5^ in our case–control data. Given its low MAF of 0.04, the gradual loss of imputation accuracy observed for rs113593463 is, therefore, more of proof than not that SNP genotype imputation is hampered by a local ME upon disease risk.

Our study also shows that particularly for ME SNPs with low MAF, the accuracy scores provided by the imputation software used may still be high even in situations where Cohen’s kappa between imputed and original SNP genotypes is low, indicative of poor imputation quality. This means that using accuracy scores as a means of quality control does not obviate the need for caution, particularly when imputing patient SNP genotypes from population controls in disease-associated gene regions where such practise is likely to render the resulting case genotypes systematically more similar to the controls. Since not all MEs may be known in the first place, negative findings of CO studies of G × E interaction should, therefore, not be over-interpreted and held too firm a proof of the absence of such effects.

Of course, the above concerns about representativeness apply to the CO and the CC study design alike, and we suspect that the effect on the power to detect G ﻿× E with the latter would be similar to that demonstrated for the CO design by our simulations. However, the consequences are more dramatic for the CO design: For one, CO studies lack controls that could potentially highlight unknown MEs in the process. Second, and more importantly, the main benefit of the CO design is an increase in power over the CC design, and this advantage may vanish unnoticed due to the subliminal reduction in imputation accuracy.

Poor genotype imputation also clearly affects the quality of the statistical analysis of G × E interactions when using a CO design. Thus, our simulation-based study of ME SNPs highlights that their G × E interaction ORs are systematically underestimated from imputed genotypes and that this effect gets more prominent when the level of LD between the target SNP and SNPs in the imputation base decreases. Consequently, the power to detect G × E interaction was found to deteriorate in a similar LD-dependent manner. Although the consequences for G × E analysis of poor genotype imputation were most serious for ME SNPs with low MAF, biased OR estimates and a loss of statistical power also became evident in the high MAF category. Moreover, when examining statistical power in relation to the local level of LD, a sudden widening of the boxplots could be noted for ME SNPs with high MAF when the *r*^2^ threshold changed from 0.6 to 0.5. Since a similar kinking occurred in this SNP category for Cohen’s kappa, we may surmise that the requirement, in CO studies of G × E interaction, of a sufficiently rich imputation base of SNPs with strong LD to the target SNP is not a question of MAF alone.

In our analyses, we considered only dominant G × E effects whereas, for most polygenic traits analyzed in GWAS so far, an additive model was most often used to study the respective main effects. With a low MAF, where the power loss was found to be most dramatic in CO studies of G × E, the difference to an additive model is, however, small. Moreover, additive models bear a risk of being less stable computationally owing to the sparseness of certain cells of the underlying G × E tables. Since we preferred to use the same genetic model for all MAF categories for the sake of comparability, we thus chose to consistently employ the more stable dominant model of G × E throughout our study.

The power assessments in our G × E simulations were all based upon a nominal significance level of 0.05 which, given that we tested multiple G ﻿× E ORs may seem inappropriate at first glance. However, for studying the impact of genotype imputation on power, the choice of significance level is of minor importance and the effects seen in our simulations are likely to be similar at more stringent significance levels. Studying the latter was, however, beyond the possibilities of our real-life genotype data because the power to detect G × E would have been low to start with, or G ﻿× E ORs should have been unrealistically high to provide reasonable power.

Interestingly, for low exposure frequency, i.e. 10% in our study, the statistical power to detect G × E interaction appeared to be systematically lower than suggested by the QUANTO software which was used to calculate the underlying simulation parameters. In fact, a median statistical power of 80% was rarely achieved. To a considerable extent, this failure may be due to the comparatively small sample size of 719 cases in our study because, at a MAF of 0.021, the QUANTO results indicated that an interaction OR of 2.95 was detectable with 80% statistical power. With a low MAF of say 0.05, however, the mean number of exposed carriers of the minor allele equals 7, which implies that more than 99.5% of the simulations were expected to yield less than 15 such cases. Hence, one likely explanation of the lower than expected power seen in our simulations is that the large sample theory underlying QUANTO (or similar software) was not taking sufficient effect at the lower end of the MAF range.

The consideration of non-genotyped SNPs may be essential to detect a particular G × E interaction, depending upon the nature and location of the genetic variants involved. Of course, such interactions could be missed if genotype imputation was omitted entirely from CO studies, but the possible pitfalls of the technique highlighted by our study need to be taken into consideration as well. It all comes down to the fact that genotype imputation requires an imputation base, i.e. a reference sample of true genotypes of nearby SNPs, and it is all too clear that the imputation quality hinges on the comparability of the imputation base and the CO sample. This limitation does not only apply to ethnicity, but to disease status as well. Cases are genetically different from the general population (usually represented by the imputation base) in regions with MEs, because this is what makes an ME an ME. Consequently, imputation-based analysis of G × E interactions in CO studies is inherently problematic, albeit to a variable extent, depending upon various factors. Thus, poor performance was shown in our study to affect target SNPs with low MAF and located in regions that are not saturated by sufficiently many SNPs in strong LD with the target SNPs. The main problem in these instances was a lack of power, rather than an inflation of false-positive results, a shortcoming that could perhaps be overcome in practice by the use of denser SNP sets or by an increase of sample size.

## Supplementary Information

Below is the link to the electronic supplementary material.

Supplementary file1 (PDF 2732 KB)

## Data Availability

The data underlying this study are available from the PopGen biobank (info@p2n-sh.de).

## Code availability

All software code used in this study is available from the first author (resch@medinfo.uni-kiel.de).
