# Supplementary material for: Genotype imputation in case-only studies of gene-environment interaction: validity and power
Source: Hum Genet. 2021 May 26;140(8):1217–28. doi: 10.1007/s00439-021-02294-z (PMC8263402; doi:10.1007/s00439-021-02294-z)
Supplement: Supplementary file 1 — Supplementary file1 (PDF 2732 KB) [file 439_2021_2294_MOESM1_ESM.pdf]

Supplementary Table 1: Target SNPs included in simulation-based G×E study.

MAF Category	Chromosome	SNP with ME					Matched SNP without ME		
		Position	ID	ME OR	ME p value	MAF	Position	ID	MAF
Low	1	67697069	rs75328060	0.39	1.61E-06	0.02	152191469	rs150298716	0.01
Low	1	67702526	rs11465804	0.43	1.64E-06	0.03	93342890	rs112043172	0.02
Low	1	67708155	rs80174646	0.40	1.81E-07	0.03	7810923	rs75455404	0.03
Low	1	67726104	rs9988642	0.44	8.67E-07	0.03	93499216	rs72730414	0.02
Low	1	67731368	rs7547569	0.44	8.57E-07	0.03	160737722	rs524276	0.02
Medium High	1	67607135	rs736197	0.63	4.75E-08	0.23	71929968	rs6703162	0.24
Medium High	1	67624304	rs12043240	0.62	1.98E-08	0.23	101543205	rs4617425	0.23
High	1	67661041	rs6656929	0.63	9.87E-08	0.27	205369075	rs1928442	0.28
High	1	67664840	rs58621044	0.63	1.05E-07	0.28	172854334	rs4916283	0.27
High	1	67665210	rs1569923	0.60	4.83E-09	0.31	92573670	rs11166163	0.31
High	1	67668970	rs6588249	1.55	4.32E-07	0.37	152599265	rs925976	0.38
High	1	67672765	rs7539625	1.61	3.26E-08	0.35	67744500	rs11209033	0.36
High	1	172838848	rs34884278	1.49	2.67E-06	0.34	198886404	rs1275161	0.34
Low	2	100576838	rs114236993	2.74	2.87E-06	0.03	181971974	rs77312863	0.03
Medium High	4	3467519	rs10022329	1.54	8.70E-06	0.16	102704134	rs6532967	0.16
Low	5	102135253	rs79045655	8.04	3.60E-09	0.02	96150110	rs80041669	0.01
Medium Low	5	40318419	rs7733749	0.64	3.08E-06	0.15	96004363	rs7704167	0.14
Medium Low	5	150174295	rs12655654	1.65	8.95E-06	0.11	26112192	rs9293221	0.11
Medium Low	5	150245724	rs10875560	1.66	3.85E-06	0.11	72493896	rs10036050	0.10
Medium High	5	40288878	rs4245975	0.66	9.31E-06	0.15	72484883	rs57125671	0.15
Medium High	5	150188072	rs10056694	1.55	5.20E-06	0.16	159849586	rs1895320	0.17
High	5	40323714	rs348595	0.68	6.34E-06	0.31	111682654	rs1019743	0.32
Low	6	34734915	rs77262732	2.24	7.82E-06	0.04	126797000	rs79353145	0.04
Medium High	6	32681992	rs3998158	0.64	2.09E-06	0.16	30388936	rs9261893	0.16
High	6	20809890	rs6902880	1.63	4.16E-08	0.41	29691019	rs1362126	0.41
High	6	28829486	rs209165	0.68	5.42E-06	0.25	29461914	rs1233490	0.26

MAF Category	Chromosome	SNP with ME					Matched SNP without ME		
		Position	ID	ME OR	ME p value	MAF	Position	ID	MAF
Low	7	75171091	rs10499824	4.39	7.70E-09	0.02	50267315	rs2366296	0.03
Low	8	11006828	rs7008658	2.71	7.52E-08	0.04	141525327	rs2231524	0.05
Low	8	11128037	rs117460861	4.14	1.07E-06	0.02	129149288	rs117302259	0.01
High	12	113031474	rs233722	1.53	5.15E-06	0.46	40532151	rs937109	0.46
Medium Low	14	98409403	rs78558838	0.58	7.67E-06	0.07	81383427	rs7144634	0.06
Low	16	50756540	rs2066845	2.55	9.63E-08	0.04	50719743	rs113593463	0.04
Low	16	50762771	rs72796367	2.18	5.96E-07	0.05	75218899	rs79206226	0.04
Low	16	50810973	rs75337140	0.27	1.93E-06	0.01	11229329	rs1540358	0.01
Low	16	50826942	rs75157714	0.27	2.12E-06	0.01	30701362	rs75843584	0.01
Low	16	50846717	rs75146978	0.41	7.38E-06	0.02	30888012	rs34640030	0.02
Medium Low	16	50827601	rs2302759	0.57	4.45E-09	0.12	75370230	rs3844219	0.13
Medium Low	16	75244425	rs117688319	2.45	9.22E-08	0.05	75420854	rs74208577	0.06
Medium High	16	50751175	rs751271	0.55	6.33E-12	0.22	81821203	rs4584816	0.22
Medium High	16	50751972	rs13332952	0.53	3.65E-13	0.23	11296285	rs59785529	0.23
High	16	50565970	rs1990623	1.84	2.09E-12	0.26	11083069	rs56231421	0.27
High	16	50661273	rs9673419	1.49	3.29E-06	0.28	11281218	rs248831	0.29
High	16	50744624	rs2066842	1.89	3.96E-13	0.44	30172627	rs13331817	0.46
High	16	50752815	rs1861757	0.65	5.96E-07	0.34	11177824	rs12708715	0.34
High	16	50766127	rs3135499	0.63	1.32E-07	0.35	11254331	rs41367	0.35
High	16	50769563	rs718226	0.62	3.52E-08	0.36	11403753	rs28671554	0.36
High	16	50798929	rs2066851	0.63	1.61E-07	0.36	30934075	rs3813020	0.36
Medium Low	20	62308612	rs2738783	0.63	1.53E-06	0.14	48599561	rs4647955	0.13
Medium High	20	62203748	rs34681475	0.63	3.55E-07	0.18	1539350	rs2250199	0.18
Medium High	20	62219740	rs3810492	0.67	7.57E-06	0.19	4055167	rs16989193	0.19
Medium High	20	62315593	rs34894559	0.67	7.41E-06	0.17	30150077	rs6060002	0.17
Medium High	20	62342654	rs2750480	0.67	9.22E-06	0.17	57597645	rs6070696	0.17
Medium High	20	62343956	rs2315008	0.65	6.19E-07	0.24	10769074	rs6032951	0.23
Medium High	20	62348460	rs4809329	0.65	5.64E-07	0.24	36266789	rs6067117	0.24

MAF Category	Chromosome	SNP with ME					Matched SNP without ME		
		Position	ID	ME OR	ME p value	MAF	Position	ID	MAF
Medium High	20	62361737	rs2427530	0.67	8.40E-06	0.17	62291008	rs6089953	0.17
Medium High	20	62362563	rs6062509	0.67	2.22E-06	0.24	51318351	rs6013509	0.24
Medium High	20	62381979	rs6089970	0.62	2.85E-07	0.16	44757213	rs3765457	0.15
Medium High	21	16838572	rs35910543	0.66	1.53E-06	0.23	44487111	rs234711	0.24
High	21	16805220	rs1736135	0.66	1.28E-06	0.36	40486507	rs2037922	0.37

MAF: minor allele frequency in cases; ME: main effect of carriership of the minor SNP allele on Crohn disease (CD) risk; OR: odds ratio. Chromosomal positions are based upon GRCh37. The ME OR was estimated by logistic regression analysis (719 CD patients, 2491 controls) as OR=anti-$\log_e(\beta_1)$, with β_1 denoting the respective regression coefficient. The ME p value corresponds to a Wald test of H_0: $\{\beta=0\}$.

Supplementary Table 2: Genotype-specific exposure probabilities (f_G) underlying G×E simulation.

Target SNP			10% Environmental Exposure Frequency			30% Environmental Exposure Frequency		
ID	ME OR	MAF	G×E OR	f_C	f_H	G×E OR	f_C	f_H
rs75157714	0.27	0.01	8.10	0.46	0.10	6.10	0.72	0.30
rs75337140	0.27	0.01	8.10	0.46	0.10	6.10	0.72	0.30
rs117460861	4.14	0.02	2.10	0.19	0.10	1.80	0.43	0.30
rs79045655	8.04	0.02	1.85	0.17	0.10	1.65	0.41	0.30
rs75328060	0.39	0.02	4.45	0.32	0.10	3.45	0.59	0.29
rs75146978	0.41	0.02	4.30	0.31	0.10	3.35	0.58	0.29
rs10499824	4.39	0.02	1.95	0.18	0.10	1.70	0.42	0.30
rs11465804	0.43	0.03	3.85	0.29	0.09	3.00	0.55	0.29
rs80174646	0.40	0.03	3.80	0.28	0.09	3.00	0.55	0.29
rs114236993	2.74	0.03	2.05	0.18	0.10	1.75	0.42	0.30
rs7547569	0.44	0.03	3.55	0.27	0.09	2.80	0.54	0.29
rs9988642	0.44	0.03	3.50	0.27	0.09	2.80	0.54	0.29
rs77262732	2.24	0.04	2.05	0.18	0.10	1.75	0.42	0.30
rs7008658	2.71	0.04	1.95	0.17	0.10	1.70	0.42	0.30
rs2066845	2.55	0.04	1.95	0.17	0.10	1.70	0.42	0.29
rs117688319	2.45	0.05	1.95	0.17	0.10	1.65	0.41	0.29
rs72796367	2.18	0.05	1.90	0.17	0.10	1.65	0.41	0.29
rs78558838	0.58	0.07	2.40	0.20	0.09	2.00	0.45	0.29
rs12655654	1.65	0.11	1.80	0.16	0.09	1.60	0.39	0.29
rs10875560	1.66	0.11	1.80	0.16	0.09	1.60	0.39	0.29
rs2302759	0.57	0.12	2.05	0.17	0.09	1.75	0.41	0.28
rs2738783	0.63	0.14	1.95	0.16	0.09	1.70	0.40	0.28
rs7733749	0.64	0.15	1.90	0.16	0.09	1.65	0.39	0.28
rs4245975	0.66	0.15	1.90	0.16	0.09	1.65	0.39	0.28
rs10022329	1.54	0.16	1.75	0.15	0.09	1.55	0.38	0.28
rs6089970	0.62	0.16	1.90	0.16	0.09	1.65	0.39	0.28
rs10056694	1.55	0.16	1.75	0.15	0.09	1.55	0.38	0.28

Target SNP			10% Environmental Exposure Frequency			30% Environmental Exposure Frequency		
**ID**	**ME OR**	**MAF**	**G×E OR**	**f_C**	**f_H**	**G×E OR**	**f_C**	**f_H**
rs3998158	0.64	0.16	1.90	0.16	0.09	1.65	0.39	0.28
rs2427530	0.67	0.17	1.85	0.15	0.09	1.60	0.39	0.28
rs2750480	0.67	0.17	1.85	0.15	0.09	1.60	0.39	0.28
rs34894559	0.67	0.17	1.85	0.15	0.09	1.60	0.39	0.28
rs34681475	0.63	0.18	1.85	0.15	0.09	1.60	0.39	0.28
rs3810492	0.67	0.19	1.85	0.15	0.09	1.60	0.38	0.28
rs751271	0.55	0.22	1.85	0.15	0.09	1.60	0.38	0.28
rs13332952	0.53	0.23	1.85	0.15	0.09	1.60	0.38	0.28
rs12043240	0.62	0.23	1.80	0.15	0.09	1.55	0.37	0.28
rs35910543	0.66	0.23	1.80	0.15	0.09	1.55	0.37	0.28
rs736197	0.63	0.23	1.80	0.15	0.09	1.55	0.37	0.28
rs6062509	0.67	0.24	1.75	0.14	0.09	1.50	0.37	0.28
rs2315008	0.65	0.24	1.75	0.14	0.09	1.50	0.37	0.28
rs4809329	0.65	0.24	1.75	0.14	0.09	1.50	0.37	0.28
rs209165	0.68	0.25	1.75	0.14	0.09	1.50	0.37	0.28
rs1990623	1.84	0.26	1.80	0.14	0.08	1.55	0.37	0.28
rs6656929	0.63	0.27	1.75	0.14	0.09	1.55	0.37	0.27
rs58621044	0.63	0.28	1.75	0.14	0.08	1.55	0.37	0.27
rs9673419	1.49	0.28	1.75	0.14	0.08	1.55	0.37	0.27
rs1569923	0.60	0.31	1.75	0.14	0.08	1.55	0.37	0.27
rs348595	0.68	0.31	1.75	0.14	0.08	1.55	0.36	0.27
rs34884278	1.49	0.34	1.80	0.14	0.08	1.60	0.37	0.27
rs1861757	0.65	0.34	1.75	0.14	0.08	1.55	0.36	0.27
rs7539625	1.61	0.35	1.85	0.14	0.08	1.65	0.37	0.26
rs3135499	0.63	0.35	1.75	0.13	0.08	1.55	0.36	0.27
rs718226	0.62	0.36	1.75	0.13	0.08	1.55	0.36	0.27
rs1736135	0.66	0.36	1.75	0.13	0.08	1.55	0.36	0.27
rs2066851	0.63	0.36	1.75	0.13	0.08	1.55	0.36	0.27
rs6588249	1.55	0.37	1.85	0.14	0.08	1.65	0.37	0.26

Target SNP			10% Environmental Exposure Frequency			30% Environmental Exposure Frequency		
**ID**	**ME OR**	**MAF**	**G×E OR**	**f_C**	**f_H**	**G×E OR**	**f_C**	**f_H**
rs6902880	1.63	0.41	1.95	0.14	0.08	1.70	0.37	0.25
rs2066842	1.89	0.44	2.05	0.14	0.07	1.80	0.37	0.25
rs233722	1.53	0.46	2.00	0.13	0.07	1.75	0.36	0.25

G×E OR: minimum gene-environment interaction odds ratio detectable with 80% statistical power in 719 CD cases, given the respective combination of MAF, ME OR and environmental exposure frequency; f_C (f_H): genotype-specific exposure probability for carriers of the minor allele (homozygotes for the major allele); for further details, see legend to Supplementary Table 1. G×E OR thresholds were calculated with QUANTO software (Gauderman 2002b).

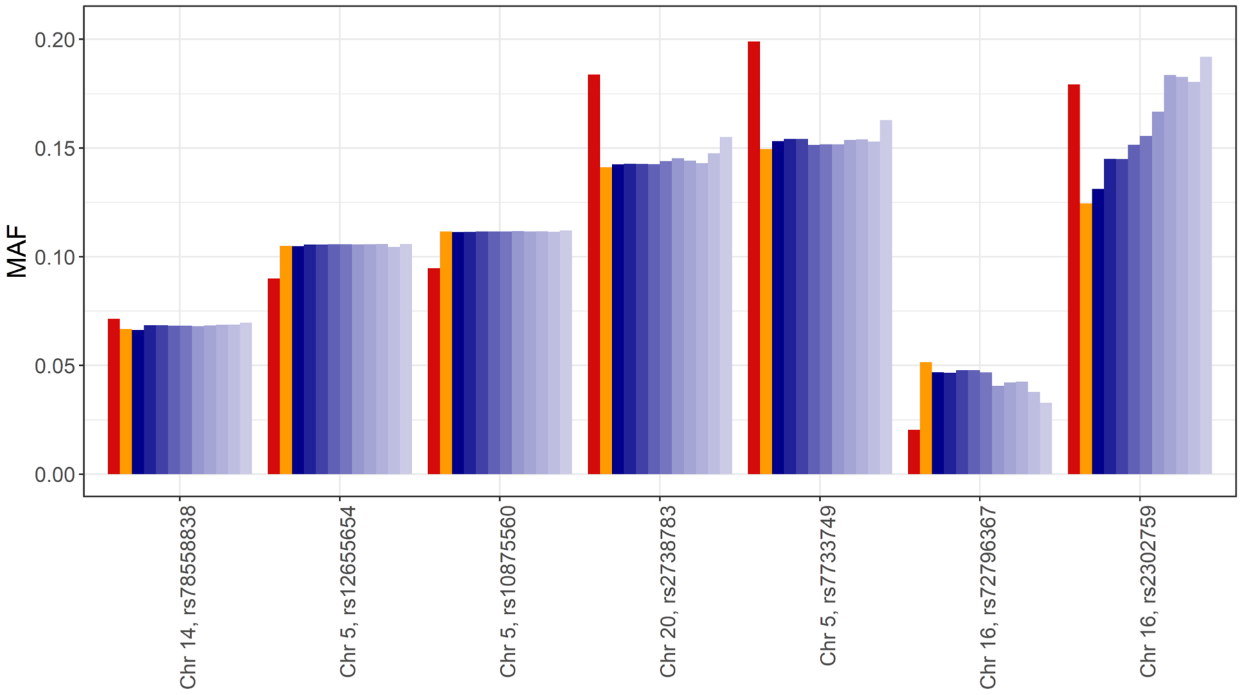

(a)

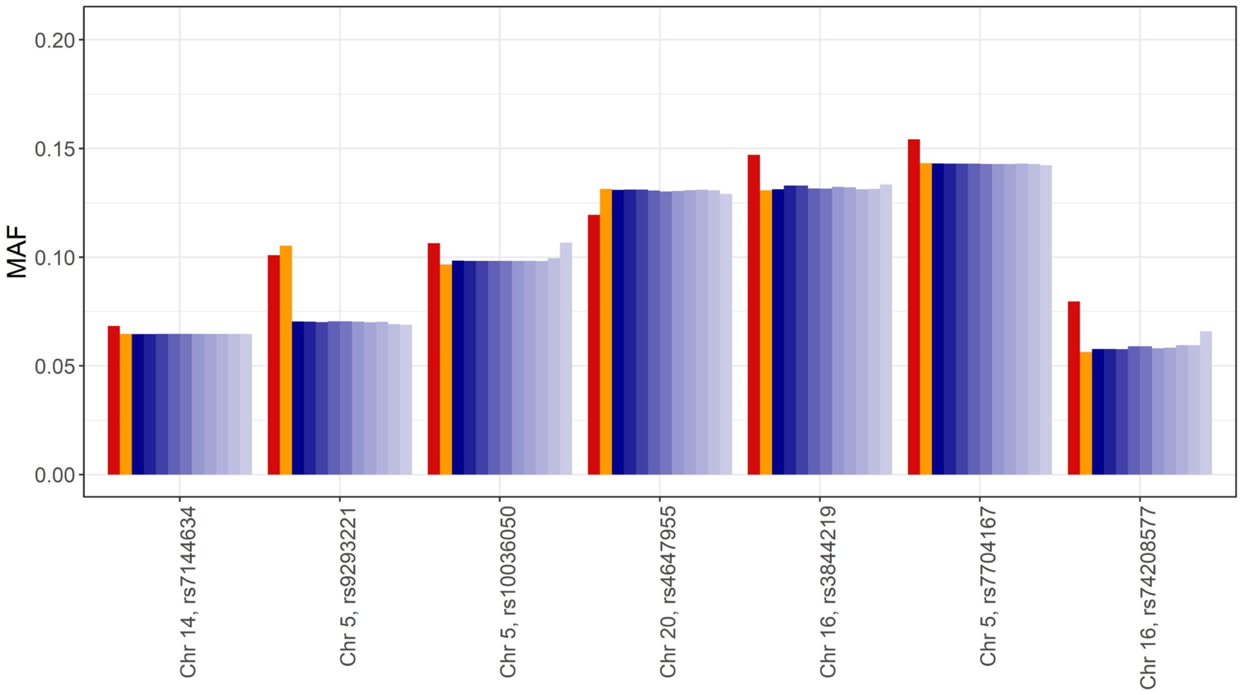

(b)

Supplementary Figure 1: MAF of real and imputed genotypes of SNPs in the medium low MAF category (0.05≤MAF<0.15). Target SNPs are grouped according to whether they had a main effect on IBD risk, i.e. showed a significant association with IBD in the study data (a), or lacked a main effect (b). The left-most bar (red) depicts the MAF of each SNP in the HRC European population data that served as the imputation base. The next bar (orange) marks the real MAF in cases. The adjacent bars (shades of blue) depict the MAF at advancing levels of LD pruning of the SNPs surrounding the target SNP, namely from $r^2<1.0$ to $r^2<0.1$ in steps of 0.1 (for details, see Methods).

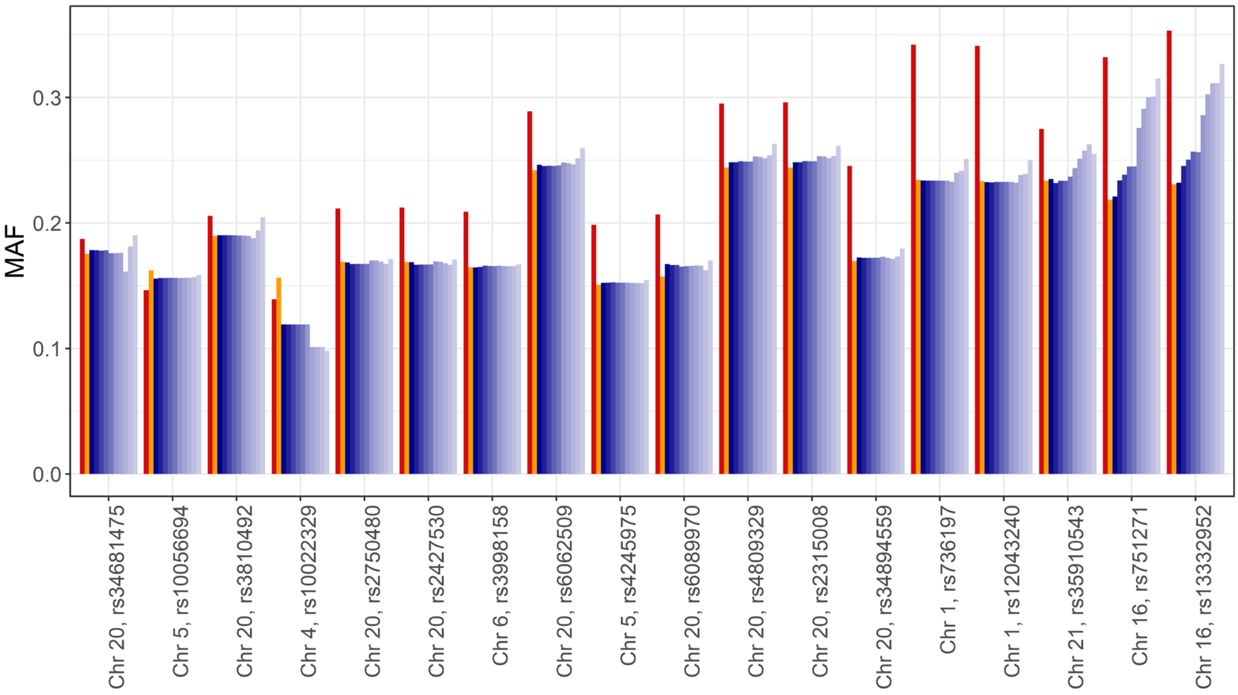

(a)

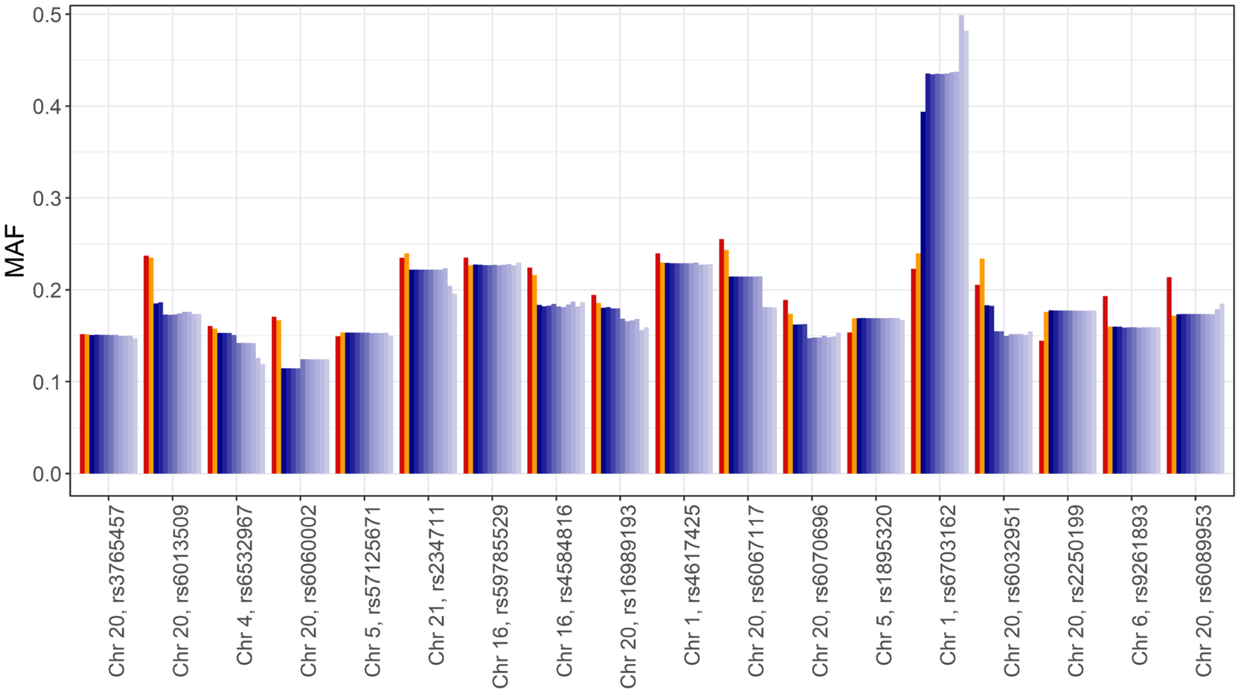

(b)

Supplementary Figure 2: MAF of real and imputed genotypes of SNPs in the medium high MAF category (0.15≤ MAF<0.25). For details, see legend to Supplementary Figure 1.

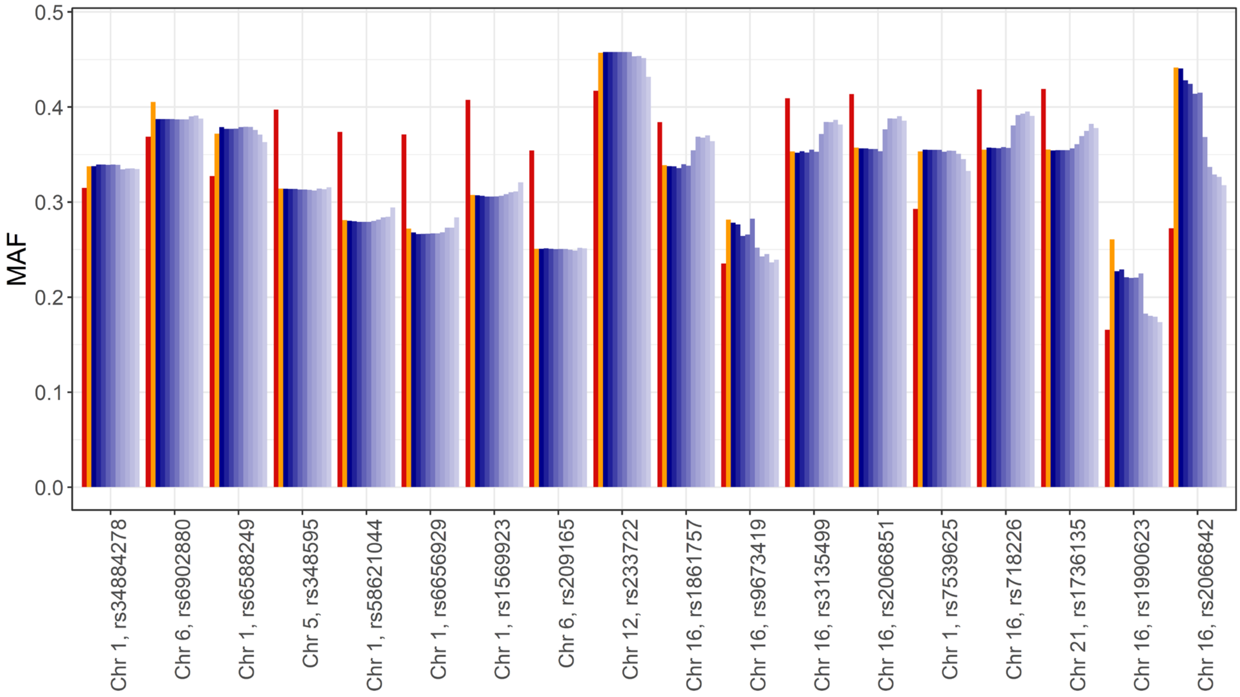

(a)

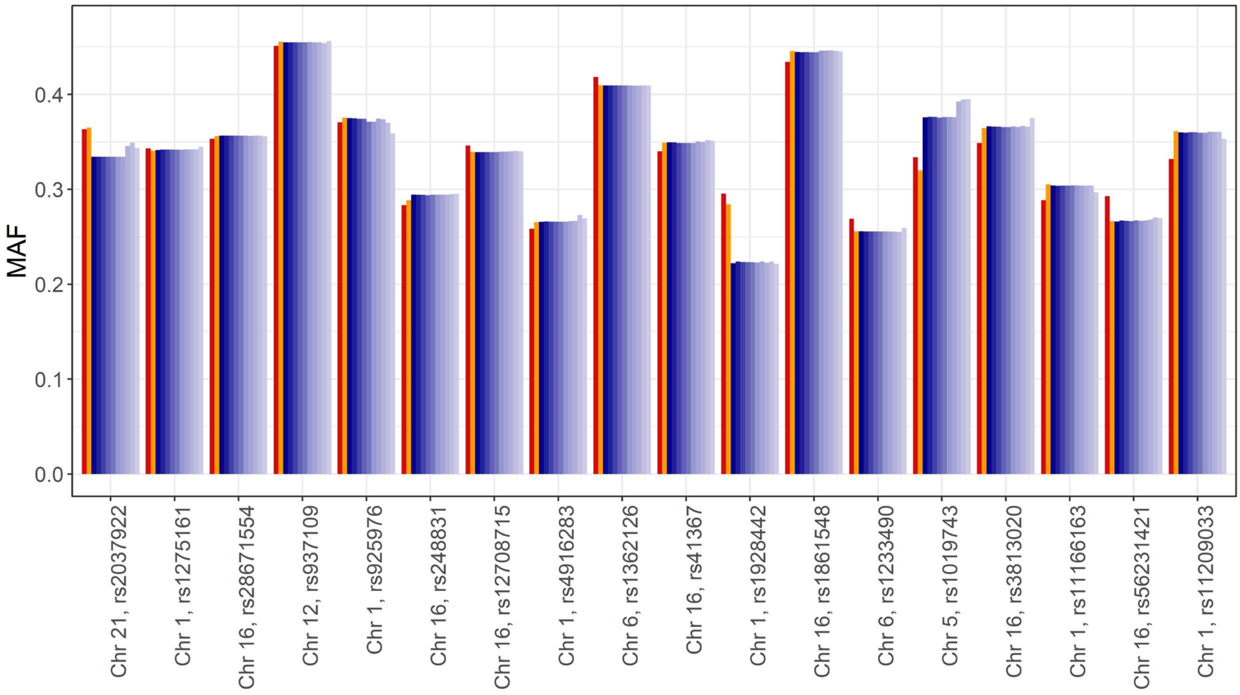

(b)

Supplementary Figure 3: MAF of real and imputed genotypes of SNPs in the high MAF category (0.25≥MAF). For details, see legend to Supplementary Figure 1.

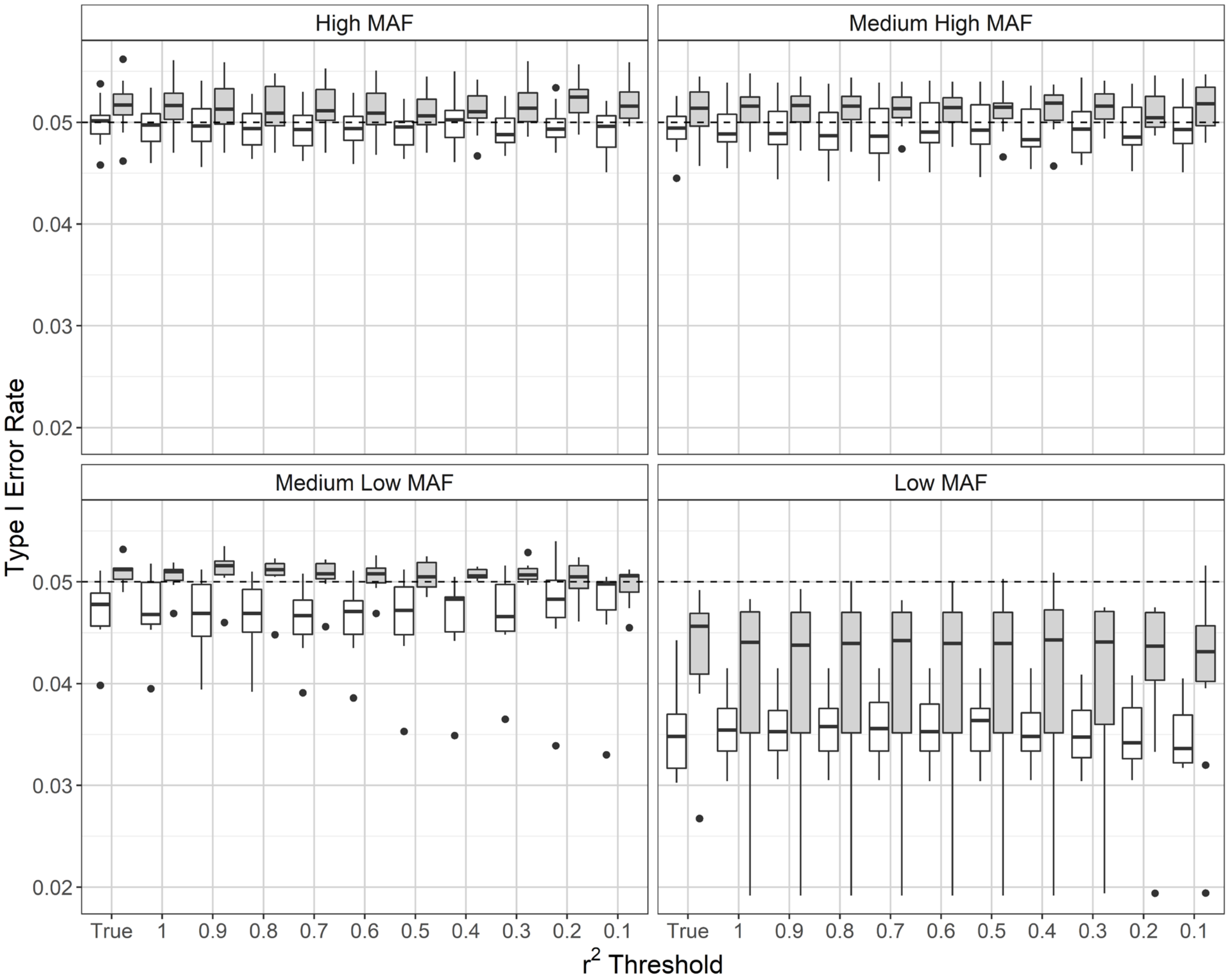

Supplementary Figure 4: Simulation-based estimates of the type I error rate of G×E interaction analysis (for details, see main text and legend to Figure 4). Horizontal axis: r^2 threshold used for LD pruning around the target SNP (for details, see main text and legend to Figure 2); True: type I error rate incurred by using the true (i.e. non-imputed SNP) genotypes. Boxes are tinted according to the environmental exposure frequency underlying the simulations (grey: 30%, white: 10%). The dashed horizontal line marks a 5% type I error rate.

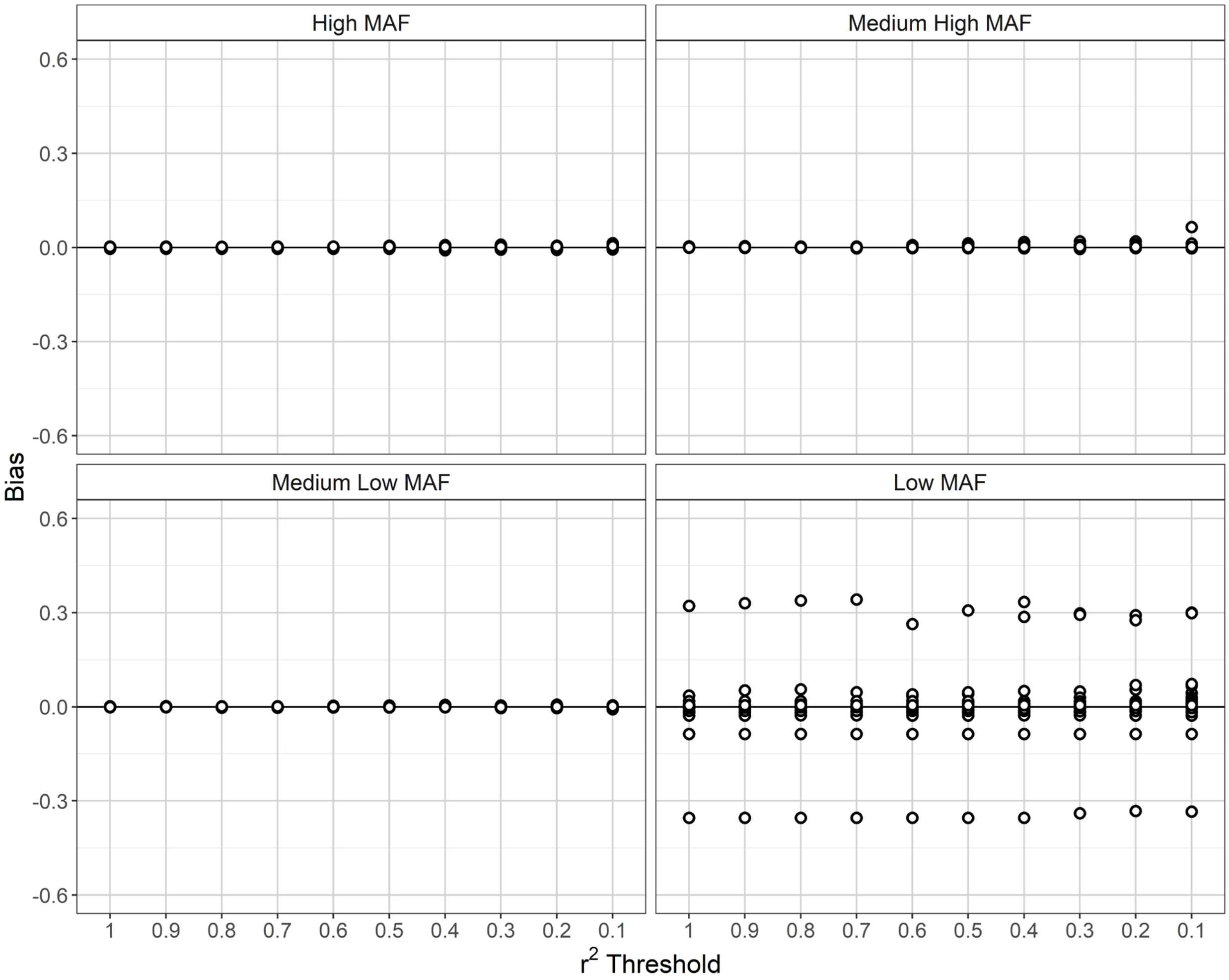

Supplementary Figure 5: Bias, under the hypothesis of no G×E interaction, of logistic regression coefficients β (i.e. logarithms of G×E interaction OR) at different levels of LD pruning. The bias was calculated as true genotype-based β minus imputation-based β.
